# Flavoprotein Fluorescence Correlation with Visual Acuity Response in Patients Receiving Anti-VEGF Injection for Diabetic Macular Edema

**DOI:** 10.1155/2018/3567306

**Published:** 2018-08-09

**Authors:** Jorge S. Andrade Romo, Giselle Lynch, Kevin Liu, Daniel Kim, Michael Jansen, Matthew G. Field, Victor M. Elner, Richard B. Rosen

**Affiliations:** ^1^New York Eye and Ear Infirmary of Mount Sinai, New York, NY, USA; ^2^Icahn School of Medicine at Mount Sinai, New York, NY, USA; ^3^Department of Ophthalmology and Visual Sciences, University of Michigan, Ann Arbor, MI, USA

## Abstract

Anti-VEGF treatment of diabetic macular edema (DME) complicating diabetic retinopathy (DR) has greatly improved structural and visual outcomes for patients with diabetes mellitus. However, up to 50% of patients are either nonresponsive or refractory to anti-VEGF treatment (no improvement in BCVA or central macular thickness (CMT)). It is believed that factors such as mitochondrial structural and functional damage, due to oxidative stress, are partially responsible for this lack of improvement. Flavoprotein fluorescence (FPF) has been shown to be a sensitive marker of mitochondrial function and has been found to correlate with the degree of diabetic retinopathy. FPF may also provide additional information regarding therapeutic response of patients receiving anti-VEGF treatment for DME. Eight patients with DR and DME with clinically significant DME (CSDME) who underwent anti-VEGF (bevacizumab) treatment were imaged before injection and at follow-up visit using FPF in addition to standard color fundus photography and OCT CMT. A strong correlation *r* = 0.98 (*p* = 0.000015) between the FPF decrease and the BCVA improvement was observed; BCVA improved as FPF values decreased. Notably, in the same patients, the correlation between OCT CMT decrease and BCVA improvement (*r* = 0.688) was not found to be significant (*p* = 0.13). These findings suggest that FPF can detect improvement in metabolic function preceding structural improvement and even with small changes in edema. Additionally, FPF may be supplementary to current diagnostic methods for earlier detection of therapeutic response to anti-VEGF treatment in patients with DME.

## 1. Introduction

Diabetic macular edema (DME), complicating advanced diabetic retinopathy (DR), may result in severe visual morbidity. Current primary therapy for DME is aimed at stabilizing damage, leaking blood vessels by administration of antivascular endothelial growth factor (VEGF) agents into the vitreous when clinically significant diabetic macular edema (CSDME) is present. In 1-year treatment studies, approximately 30 to 40% of patients who receive anti-VEGF treatment improve 3 or more Snellen lines of vision [1–3]. However, up to 50% of patients either are not responsive or become refractory to anti-VEGF agents, with many of these eyes salvaged with intravitreal corticosteroids [1, 4]. The variability in DME responses to anti-VEGF therapy suggests that DME is the result of multiple factors underlying the pathogenesis of DR. Anti-VEGF agents may stabilize vascular leakage and reduce edema but may not address other factors which contribute to CSDME or adversely impact best corrected visual acuity (BCVA), the most important endpoint for DME treatment. In fact, recent large, multicenter studies have shown that the best anti-VEGF treatments improve diabetic retinopathy severity scores (DRSS), but do not result in statistically significant improved BCVA [5]. Other factors that are likely to underlie reduced BCVA in DME include oxidative stress, due to combinations of apoptotic and proinflammatory signaling, genomic and mitochondrial DNA alterations, due to oxidative damage and other abnormal molecular signaling, mitochondrial structural and metabolic damage, loss of neuroprotective and growth factor cytokines, and the direct effects of hyperglycemia on impairing cell survival [6, 7]. Anti-VEGF therapy may indirectly affect some of these factors, but is not aimed at them specifically, as are several other new therapies currently in development and testing [6].

The most widely used clinical method for monitoring anti-VEGF treatment effect today is optical coherence tomography (OCT). OCT is capable of measuring central macular thickness (CMT), demonstrating fluid accumulation within and beneath the retina, and detecting anatomic disruption of the various layers of the retina, all features which have been used for measuring anti-VEGF therapy efficacy [8]. OCT is a structural tool which only indirectly measures retinal metabolic dysfunction, evidenced by accumulation of fluid which disrupts normal retinal architecture [4, 8, 9]. While quantitative reductions in edema and restoration in structure seen with OCT can detect anti-VEGF effect, these show variable correlation with BCVA presumably because other factors are relevant to visual functioning [8, 10, 11]. OCT monitoring is also of limited usefulness in monitoring therapeutic response in DME patients with mild to moderate reductions in BCVA and minimal retinal thickening [12]. Thus, OCT, while helpful in identifying retinal edema, falls short in detecting the prestructural aspects of oxidative stress and other contributing pathogenetic factors.

Flavoprotein fluorescence (FPF) is a promising biomarker for the monitoring of retinal metabolic integrity [13, 14]. Studies on primary human retinal cell cultures and ex vivo neurosensory human retinal tissue showed FPF to be a sensitive marker of mitochondrial dysfunction and early apoptotic responses due to oxidants and other proapoptotic stimuli. Specifically, increasing levels of oxidative stress resulted in a progressive rise of FPF levels, which directly correlate with reduction in the mitochondrial membrane potential and increased risk of caspase-3 cleavage and apoptosis [14–16]. DR is due to a complex interplay of multiple pathways leading to increased aldose reductase, protein kinase C, and hexosamine pathway activities, glycation of proteins, nitrosylation, and damage to both nuclear and mitochondrial DNA [17–19]. All of these perturbations may be linked to mitochondrial depolarization, dysfunction, and damage with the associated generation of superoxides and other free radicals [17, 18, 20, 21]. Mitochondrial dysfunction is recognized to be at the pathogenetic core of inflammation, apoptosis, and response to hyperglycemia in DR. [6, 7, 17–19]

FPF would appear to be an ideal biomarker of DR-associated mitochondrial dysfunction and its therapeutic mitigation, because it directly measures impairment of electron transport and ATP production that is facilitated by flavoproteins [14, 15]. Under disease conditions, flavoproteins are predominantly in an oxidized rather than physiologically reduced electronic states, their electrons susceptible to blue light excitation that results in the rapid (<5 nanosecond) emission of green FPF. When adapted for imaging of human eyes, retinal FPF was increased in diabetic humans compared with age-matched controls, proportional to disease severity and duration of disease [22]. Humans with mild background nonproliferative diabetic retinopathy (NPDR) had significantly higher FPF values compared to diabetic humans without retinopathy. Patients with more severe proliferative diabetic retinopathy (PDR) showed even higher FPF values than age-matched patients with mild NPDR. Moreover, the degree of retinopathy correlated better with FPF than serum hemoglobin A1c [22].

The monitoring of DME and its treatment could potentially be improved by directly measuring key mitochondrial metabolic alterations central to DR pathogenesis, which may reflect earlier alterations and response to therapy [12]. FPF has the potential to provide improved monitoring of the therapeutic earlier than current methods of clinical monitoring [22]. Such therapies aimed at reduction of oxidative stress, improved neuroprotection, and the salutary effects of growth factors are difficult to assess with current imaging techniques [18, 21]. Herein, we report a pilot study in which FPF was used to monitor the therapeutic effect of anti-VEGF treatment in patients with CSDME in addition to conventional fundus photography, BCVA, and OCT imaging.

## 2. Materials and Methods

### 2.1. Study Population

This study was conducted at the New York Eye and Ear Infirmary of Mount Sinai. The study protocol was approved by the Institutional Review Board and adhered to the tenets of the Declaration of Helsinki. Written informed consent was obtained from all participants prior to imaging.

Inclusion criteria were the diagnosis of DME with CSDME requiring treatment with bevacizumab (Avastin) intravitreal injection, phakia, and BCVA ≥ 20/200. Exclusion criteria included: refractive surgery; ≥grade 3 nuclear sclerotic, cortical, or posterior subcapsular cataract graded using the lens opacity classification system III; uncontrolled hypertension; systemic inflammatory disease; and retinal or vitreous hemorrhages obscuring the macula.

All subjects were classified with severe NPDR or PDR with DME graded by a trained retinal specialist according to Early Treatment Diabetic Retinopathy Study (ETDRS) criteria. Recent medical history and systemic and ophthalmic medications were recorded. Prior to bevacizumab injection, all subjects underwent complete ophthalmic examination including BCVA, Tono-Pen tonometry (Reichert Technologies, Depew, NY), pupillary dilation using tropicamide 1% eye drops administered twice at 5-minute intervals, slit-lamp biomicroscopy, fundus examination, fundus photography (Optos, Marlborough, MA), SD-OCT (Spectralis, Heidelberg Engineering Inc., Heidelberg, Germany), and FPF metabolic imaging (OcuMet Beacon, OcuSciences Inc., Ann Arbor, MI). Subjects completed the same imaging protocol during all follow-up visits.

### 2.2. Fundus Photography

All patients underwent fundus photography using an Optos imager after pupillary dilation.

### 2.3. OCT Image Acquisition

OCT scans were acquired using a Spectralis imager. A 20 × 15 raster scan comprised of 37 B-scans centered on the fovea was taken for each eye. A circular annulus centered on the fovea, of inner diameter 1 mm and outer diameter 3 mm, was used to calculate the retinal thickness values for central (macula), superior, inferior, nasal, and temporal retinal regions.

### 2.4. Metabolic Image Acquisition

Metabolic analysis of the mitochondrial function of the macula was assessed by measuring average central macular retinal FPF scores for each eye using the OcuMet Beacon [23]. The OcuMet Beacon is a customized fundus camera with special 467 nm excitation and 535 nm emission filters, attached back-illuminated electron-multiplying, charge-coupled device (EMCCD) camera, and attached computers with proprietary software.

The excitatory blue light in the OcuMet Beacon is well below the International Electrotechnical Commission's maximum permissible energy levels at 0.01 of the maximum light intensity allowed at the retina. The device is classified under the safest laser/light-emitting diode (LED) category type I, a nonsignificant risk device designation.

After pupillary dilation, the OcuMet Beacon was used to capture four to six FPF readings, by inducing 30 ms 467 nm excitation flashes focused at the macula of the retina. The depth of focus of the imager results in capture of FPF from all retinal layers in the central 13° diameter circular field of the macula, centered on the fovea. Images of low quality, including those out of focus, off-center, or with artifacts, were excluded from the analysis, obtaining ≥4 good quality images for each eye at each imaging session. The images were stored as 512 × 512-pixel 16-bit grayscale TIFF files and were analyzed with proprietary software to obtain FPF scores. To obtain the average FPF scores for each eye at each imaging session, the analysis used 4 images of each eye at each session. Imaging sessions were ≤5 minutes per eye.

### 2.5. Statistical Analysis

All consecutive patients with complete sets of prebevacizumab injection and postinjection data and 2 patients lacking only postinjection OCT data were included. Two-tailed *t*-tests were performed to assess the significances of the changes of BCVA, OCT CMT, and FPF average values before and after bevacizumab injection using SPSS (IBM Analytics, IBM Corporation, Armonk, NY, USA). Pearson correlation coefficients (PCC) were calculated to determine the associations between BCVA and average FPF changes, BCVA and OCT CMT changes, and OCT and FPF changes before and after anti-VEGF treatment. *p* values < 0.05 were considered statistically significant for all tests. For PCC, *r* ≤ −0.7 or ≥0.7 was considered to indicate a strong linear correlation.

## 3. Results

**Figure 1:**
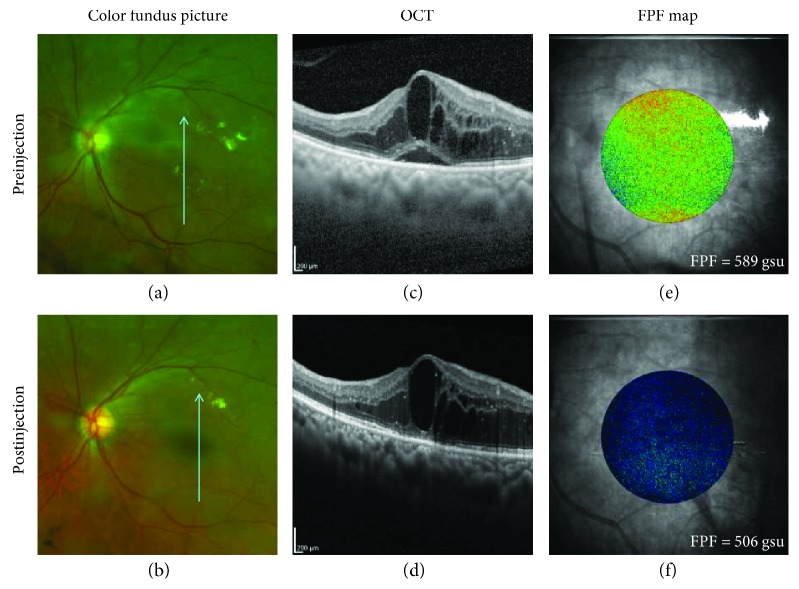
Subject 008, 65 y/o Hispanic male diagnosed with PDR and CSME. (a, b) Color fundus picture shows macular edema, hard exudates, and hemorrhages. (c) OCT CMT preinjection (574 *μ*m); (d) OCT CMT postinjection (541 *μ*m). (e) FPF map preinjection shows an increase in green-yellow coloring suggesting greater mitochondrial dysfunction (oxidative stress) compared to the (f) blue-green postinjection FPF map. gsu = grayscale units.

Demographic data for all subjects is presented in Table 1. Complete data sets (except for OCT measurements of 2 eyes) were available from 8 of 11 patients with DME, who underwent anti-VEGF treatment for CSDME (Table 2) and for 1 patient, a 60-year-old woman with DME, but not CSDME, who had anti-VEGF treatment and had a complete data set. Statistical analyses were performed on 8 subjects (6 men and 2 women; mean age 58 years, range 40–73), all of whom had type 2 DM and DME complicating PDR (5 eyes) and severe NPDR (3 eyes). Clinically significant CSDME was present in all 8 patients based on ETDRS criteria. Fundus photographs, OCT scans, and FPF maps represent the changes before and after anti-VEGF injection (Figure 1). All BCVA, OCT CMT, and FPF measurements pre- and postinjection are stated in Table 2. The percentage differences between pre- and postmeasurements are displayed in Table 3.

OCT CMT pre- and post-anti-VEGF therapy was reduced (improved) in 5 of the 6 eyes for which complete OCT data was available (Table 2). The pre- and posttreatment CMT were significantly different (*p* = 0.034: Table 4) for the 6 patients. Discrepancies between BCVA and OCT differences were most evident in 2 eyes with 20/20 or 20/25 vision and lesser degrees of macular thickening. The disparity between statistical significance for BCVA and OCT CMT values before and after anti-VEGF treatment is not surprising as OCT CMT is known to not correlate with BCVA in a substantial minority of eyes with DME.

As shown in Table 2, 5 of the 8 patients had better BCVA, 3 worse BCVA, and one unchanged BCVA after anti-VEGF treatment. Overall, the BCVA logMAR difference pre- and postinjection was not statistically significant (*p* = 0.982: Table 4), consistent with the known variability of DME responses to anti-VEGF therapy.

Average FPF intensity values improved in 5 of the 8 eyes (Table 2) after anti-VEGF treatment. As in the case of BCVA, differences before and after treatment were not statistically different (*p* = 0.289: Table 4). However, all eyes with improved BCVA also showed improve FPF, whereas all with reduced BCVA also showed elevated (worse) FPF (Table 3).

**Figure 2:**
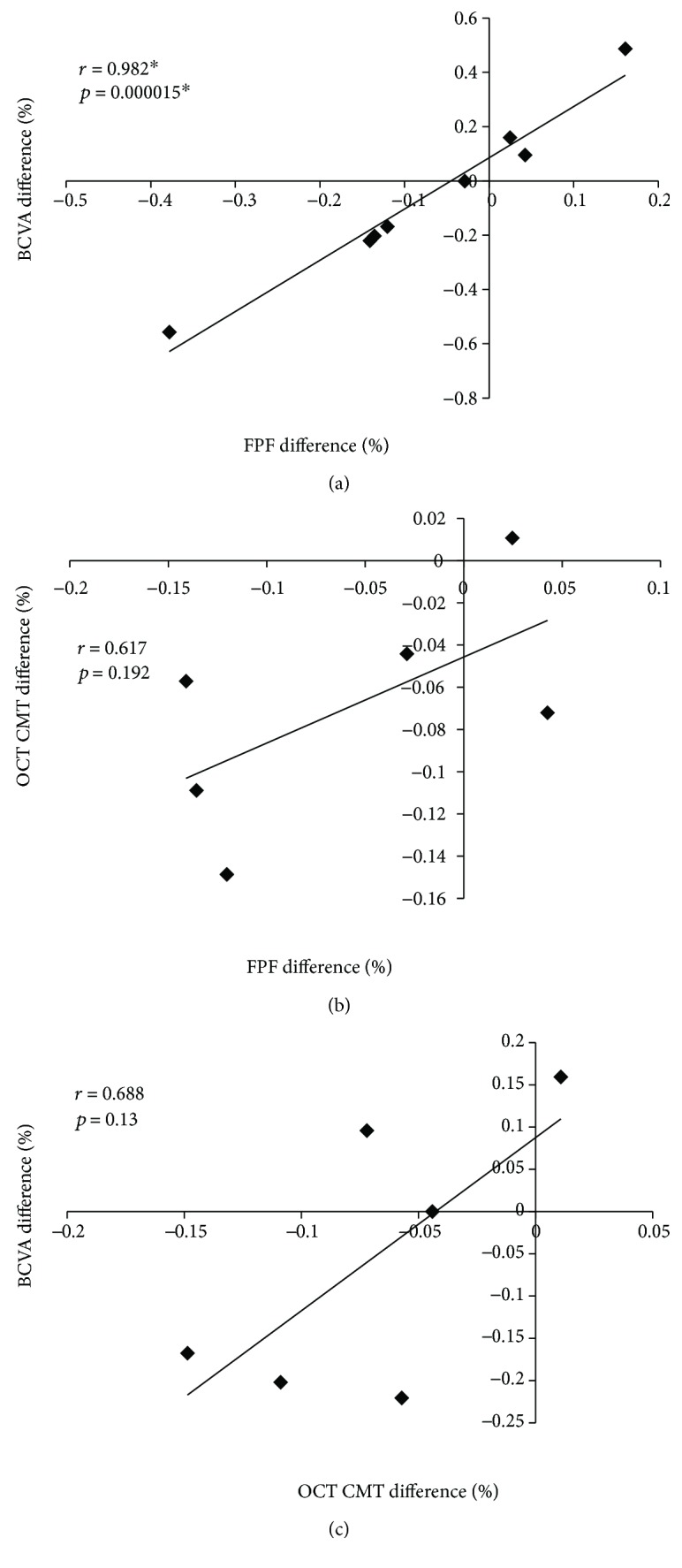
Scatter plot of Pearson's correlation coefficient from: (a) FPF difference (%) versus BCVA difference (%); (b) FPF difference (%) versus OCT CMT difference (%); (c) OCT CMT difference (%) versus BCVA difference (%). ^∗^ indicates *r* > 0.7 and *p* < 0.05.

Correlation between percentage difference in BCVA and FPF before and after treatment was highly statistically significant (*r* = 0.982, *p* = 0.000015: Figure 2(a)) for the 8 eyes. In contrast, correlation between percentage difference in FPF and OCT CMT before and after treatment did not reach statistical significance in the 6 eyes (*r* = 0.617, *p* = 0.192: Figure 2(b)). Correlation between difference percentage in BCVA and OCT CMT before and after anti-VEGF treatment likewise did not reach statistical significance (*r* = 0.688, *p* = 0.13: Figure 2(c)) for the 6 eyes for which complete data were available.

The patient who had DME, but not CSDME and underwent anti-VEGF treatment had improvement of BCVA from 20/30 to 20/25, reduction in OCT CMT of 2.4% and FPF reduction (improvement) of 52% (data not shown).

## 4. Discussion

This is the first study, to our knowledge, assessing the treatment of human DR by sequential FPF analysis of the same eyes before and after anti-VEGF treatment. Despite a small sample size, this pilot study showed a statistically significant correlation between improvement in BCVA and reduced (improved) FPF values (*r* = 0.982, *p* = 0.000015: Figure 2(a)) in 8 eyes that underwent anti-VEGF treatment of CSDME.

Previously, our FPF method was used in several cross-sectional human studies in which retinal metabolic stress of eyes with various retinal and optic nerve diseases was compared to the levels of metabolic stress in eyes of age-matched, human control individuals. An early study compared FPF of eyes of control individuals and of diabetic individuals without DR, with mild (background) NPDR, and with PDR [22]. That study showed increased FPF in diabetic eyes without DR compared to age-matched controls with progressive increasing FPF in eyes with mild NPDR and even higher FPF in eyes with PDR. Moreover, the presence and severity of NPDR and PDR correlated better with FPF than HbA1c. Most recently, a cross-sectional study of macular metabolic stress, as measured by FPF, was performed on age-matched control individuals without eye disease, patients with ocular hypertension, and patients with primary open-angle glaucoma (POAG) [23]. FPF was found to be elevated in eyes with ocular hypertension and further elevated in the remaining inner retinal tissue of eyes with POAG, when adjusted for inner retinal thickness as measured by OCT. The notion that retinal macular metabolic stress is likely to be present in POAG is supported by another study in which severe POAG eyes were treated with a proprietary nutrition supplement rich in antioxidants [24]. Eyes of supplement-treated patients exhibited statistically significant improvement in retinal FPF compared to eyes of patients not receiving supplement.

The primary goal of ophthalmic treatment of retinal and optic nerve diseases is to optimize retinal macular function, particularly BCVA. Current functional measures, besides BCVA, include testing of visual field, color vision, pupillary responses, recovery following macular light stress, electroretinography, electrooculography, and visual evoked potential. Each of these tests suffers from various combinations of subjectivity in acquisition or interpretation, need of highly skilled personnel for administration, expense, and prolonged test time. Current structural measures, though more poorly correlated with BCVA, include photography, OCT, fluorescein angiography, and ultrasonography. These measures generally demonstrate structural changes after advanced disease or after enough cell loss has occurred to alter structure to an observable level. In addition, amelioration of these measures after treatment often trails visual improvement. As a group, except for OCT, they exhibit many of the same drawbacks of the functional tests.

BCVA measurement is the most clinically relevant functional measurement of retinal health upon which clinicians rely. Our results suggest that FPF is an objective, rapid, and cost-effective functional measure that closely correlates with BCVA and correlated better than other tests used in this study, including OCT. Improvements in FPF and BCVA, but not OCT CMT, occurred in anti-VEGF treated eyes with good vision and lesser degrees of CMT, possibly because OCT resolution is less when CMT is minimal or because FPF detects improved metabolic function independent of small degrees of edema. Thus, our data suggests that FPF may be used as a secondary measure to assess the effect of anti-VEGF therapy for DME that is initially nonresponsive or becomes refractory to therapy after numerous injections [1–3].

FPF has the ability to detect alterations due to any pathophysiologic mechanism that impacts mitochondrial function and permits detection of subclinical disease and its improvement by treatment. FPF signal intensity corresponds to the proportion of FAD molecules in oxidized rather than in reduced electronic states, rendering lower energy electrons susceptible to blue light excitation. As FPF can only be elicited from living cells, its intensity is increased when mitochondria are damaged metabolically by aging or disease, but cannot be elicited from cells that are in the final stages of cell death or dead. Increased FPF signal intensity correlates closely with the loss of normal mitochondrial function and decreases in response to therapies which remedy dysfunction. During aging, light, oxygen, and high turnover of lipid-rich cellular membranes combine to produce chronic, low levels of injurious reactive oxygen intermediates (ROI), bioreactive lipids, lipid peroxides, and nitric acid that directly damage the mitochondria and upregulate p38 mitogen-activated protein kinases (MAPK) and subsequently NF-*κ*B. These in turn induce proinflammatory cytokines, such as IL-1*β*, TNF-*α*, MCP-1, and IL-6, which further increase ROI [17–19]. The p38 MAPK pathway is particularly active in astrocytes and microglia of the neurosensory retina and macrophages and retinal pigment epithelial (RPE) cells at the outer retina-blood barrier [17, 19]. These processes, mitigated by endogenous antioxidants and other anti-inflammatory molecules, are likely to be the principal causes of small and progressive increases in the FPF signal observed with aging.

In diabetic retinopathy, these processes are greatly increased. Hyperglycemia, activation of protein kinase C (PKC), and advanced glycation end products (AGE) increase ROI and stimulate p38 MAPK, NF-*κ*B, and proinflammatory cytokine production [17–19]. The mitochondrial damage that results from these potent diabetic pathological mechanisms results in profound losses of mitochondrial membrane potential (Ψ) that underpins diabetic retinal cell apoptosis and cell death [21]. The dysregulated production of proinflammatory cytokines and circulating oxidatively damaged molecules, including lipoproteins, induce a state of chronic inflammation intravascularly as well as in retinal tissue mediated in part by the aforementioned cytokines and IL-1*α* and IL-8 as well as leukocyte adhesion molecules [17–19]. The result is leukostasis and leukocyte activation and infiltration at the blood-retina barrier with leukocyte participation in ROI and proinflammatory cytokine production. These mechanisms are those that increase FPF signal in diabetics soon after the onset of diabetes before any retinopathy can be observed by any morphologic imaging of cellular dropout rather than measures of dynamic alterations in function [23].

Hypoxia that results from microvascular damage induces the expression of hypoxia-regulated cytokines and growth factors, including VEGF, which promotes neovascularization and causes breakdown of the inner and outer blood-retina barriers [17]. Interstitial tissue edema due to the action of VEGF increases tissue hypoxia and metabolic stress by impairing oxygenation and metabolite transport. Thus, endogenous VEGF has the capacity to increase hypoxia, which in turn increases ROI and ROI-induced NF-*κ*B as well as directly inducing TNF-*α* and IL-6 [25]. Conversely, anti-VEGF therapy may be expected to reduce metabolic stress and FPF intensity as we observed in this study.

FPF is elevated in eyes of diabetics without clinical DR [22] and in eyes with ocular hypertension [23] prior to optic disc or visual field alteration. In this study, significantly improved FPF and clinically improved BCVA were detected in the eye of a 60-year-old woman with DME, but without CSDME, who nevertheless improved with anti-VEGF treatment. Their findings suggest that mechanisms not due to CSDME per se may improve with anti-VEGF treatment and be detected by FPF imaging [6]. Noninvasive and rapid FPF diagnostic imaging, in conjunction with OCT, OCT-angiography, and other assessments, may become a useful way to monitor response to treatment in DR with edema.

We found that eyes receiving the first of three scheduled bevacizumab injections had greater percentage decrease in FPF and greater improvement in BCVA compared to eyes after their third injection. Eyes displayed the greatest decrease in FPF, reduced mitochondrial oxidative stress, 7 to 28 days after anti-VEGF treatment. These findings provide the opportunity to further investigate short and long-term mechanisms of anti-VEGF action and may provide guidance regarding when to combine anti-VEGF treatment with other therapies or to change to other therapy, such as intravitreal corticosteroids [2, 6, 26].

It is important to note the limitations to this study. The findings from our small sample of human eyes needs to be corroborated by additional studies involving a larger number of eyes/patients. Notably, limited information about previous injections by other providers was available; this could conceivably affect the recorded number of injections the patient had previously received. Moreover, individual adherence to therapeutic lifestyle choices and glucose control could impact on the effectiveness of bevacizumab injection, altering our ability to correlate oxidative stress response to anti-VEGF therapy. Baseline FPF values of each patient were dependent on his or her age and disease state. In this study, the FPF values measured before and after anti-VEGF treatment were designed to measure the change in metabolic stress in response to therapy. Lastly, the FPF instrument is optimized for central macular imaging. FPF imaging of other regions of the retina will be assessed in future studies.

## 5. Conclusion

This pilot study using flavoprotein fluorescence (FPF), a new dynamic measure of mitochondrial function, to monitor CSDME treated with anti-VEGF therapy shows promise as an objective early indicator of therapeutic response, correlating with changes in BCVA which appear to precede those in OCT CMT.

Future studies will expand upon FPF as a functional biomarker for diabetic treatment throughout a wider range of diabetic retinopathy severity groups and explore its value in monitoring a variety of treatment regimens.

## Figures and Tables

**Table 1:** Demographic data.

Subject ID	Sex	Age	Eye
001	M	57	OD
006	F	73	OS
008	M	65	OS
007	M	58	OS
011	M	54	OD
003	M	64	OS
028	F	57	OD
026	M	40	OD

**Table 2:** Measurements pre- and postinjection. For each subject, all preinjection measurements were taken on the day of injection, and postinjection measurements were taken at a follow-up visit days later. BCVA = best corrected visual acuity; FPF = flavoprotein fluorescence; OCT = optical coherence tomography; CMT = central macular thickness; gsu = grayscale units.

Subject ID	Preinjection BCVA	Postinjection BCVA	Preinjection OCT CMT (*μ*m)	Postinjection OCT CMT (*μ*m)	Preinjection FPF (gsu)	Postinjection FPF (gsu)	Postinjection follow-up visit (days)	Injection position in current series
001	20/50	20/30	371.8	**NA**	437	272	7	2
006	20/150	20/100	582.2	518.8	181	156	14	1
008	20/100	20/70	574	541.2	589	506	28	3
007	20/60	20/50	392.8	334.4	574	505	56	1
011	20/150	20/400	594.4	**NA**	490	569	56	1
003	20/80	20/100	540	545.8	465	477	129	3
028	20/20	20/25	319	296	423	441	27	1
026	20/20	20/20	344.6	329.4	312	303	39	3

**Table 3:** Percentage of difference in measurements from pre- to postinjection. LogMAR = logarithm of the minimum angle of resolution.

Subject ID	BCVA logMAR difference (%)	OCT CMT difference (%)	FPF difference (%)
001	−55.7	**NA**	−37.8
006	−20.2	−10.9	−13.6
008	−22.1	−5.7	−14.1
007	−16.8	−14.9	−12.0
011	48.7	**NA**	16.1
003	15.9	1.1	2.5
028	9.6	−7.2	4.3
026	0.0	−4.4	−2.9

**Table 4:** *p* values of paired sample *t*-test between pre- and postinjection measurements. ^∗^ indicates *p* < 0.05.

Group analysis	*t*-test *p* value
Pre-FPF versus post-FPF	0.289
Pre-BCVA versus post-BCVA	0.982
Pre-OCT CMT versus post-OCT CMT	0.034^∗^

## Data Availability

The data used to support the findings of this study are available from the corresponding author upon request.
